# Pleural Effusion: A Rare Presentation of Mature Teratoma in a Young Patient

**DOI:** 10.7759/cureus.15550

**Published:** 2021-06-09

**Authors:** Mubashar Iqbal, Hira Yousuf, Zara Majeed, Muhammad Zohaib, Ashish Mishra, Muhammad M Amjad, Ali Hussain

**Affiliations:** 1 Respiratory Medicine, Sheffield Teaching Hospitals NHS Foundation Trust, Sheffield, GBR; 2 Oncology, Pinderfields General Hospital, Wakefield, GBR; 3 Medicine, Nottingham University Hospital, Nottingham, GBR; 4 Internal Medicine, Hull Royal Infirmary, Kingston Upon Hull, GBR; 5 Acute Medicine, Hull Royal Infirmary, Kingston Upon Hull, GBR; 6 Endocrinology, Hull Royal Infirmary, Kingston Upon Hull, GBR

**Keywords:** mature teratoma, mediastinal mass, transudative pleural effusion, large pleural effusion, extragonadal germ cell tumor

## Abstract

Mediastinal masses always present a diagnostic challenge to clinicians and radiologists. There are wide varieties of pathologies ranging from benign to malignant conditions. Teratomas are one of the rare causes of mediastinal tumors. In this case, we report a young male who presented to the emergency room with acute pleuritic chest pain. The chest X-ray showed massive right-sided pleural effusion. Subsequently, bedside chest ultrasound ruled out septations and helped drain the fluid. The pleural fluid analysis demonstrated transudate chemistry. A computerized tomography (CT) of the chest was performed, revealing a complex anterior mediastinal mass suspected of Mature Teratoma. The tumor was surgically removed in its entirety, and pathology confirmed it a mature teratoma. The patient remained asymptomatic on postoperative follow-up.

## Introduction

Teratomas are germ cell tumors with well-differentiated tissues from all three embryonic layers. They are generally gonadal in origin yet can seldom arise from tissues outside gonads and are termed “Extra-gonadal Teratomas”. The mediastinum is the most well-known extra-gonadal site, and the majority of the teratomas at this location are benign. Teratomas are more common in the young population and have equal gender distribution. Mostly they are asymptomatic, but sometimes they present with symptoms from compression of surrounding structures. Pleural effusion is a rare presentation of mature teratoma with only a few case reports in the literature.

## Case presentation

A 25-year-old male presented to the emergency department (ED) with a two-day history of right pleuritic chest pain. The pain was in the centre of the chest and non-radiating. It was gradual in onset, dull in character, and 2/10 in intensity. There were no aggravating or alleviating factors. Furthermore, there was a history of worsening shortness of breath over a period of five months. The patient also complained of orthopnea needing two pillows but without paroxysmal nocturnal dyspnea. The patient declined a history of cough, fever, rigors, chills or hemoptysis. The patient also denied any history of recent travel, legs swelling or pain. There was no history of skin rash, joint pain or frothy urine and no history of trauma to the chest. He had a good appetite, and his weight remained stable over time. The patient was a university student and had no history of exposure to industrial fumes or dust; specifically, there was no history of asbestos exposure. He was a light smoker with a history of fewer than five packs a year. There was no family history of lung disorders or malignancy. Past medical history was unremarkable.

On initial assessment in ED, he was comfortable while sitting. Observations were normal with blood pressure 110/70 mmHg, heart rate 95 beats per minute (bpm), respiratory rate (RR) 16, and saturation of oxygen (O2) 95% on room air. He was afebrile with a temperature of 36.8˚C. There was no clubbing or lymphadenopathy.

He had decreased breath sounds at the right base on chest examination with a stony dull percussion note. Cardiovascular examination was normal. Moreover, there were no palpable abnormalities in the bilateral testis. The rest of the systemic examination was normal.

His baseline blood investigations, as shown in Table 1 were unremarkable except for a high C-reactive protein (CRP) of 80 mg/L (normal range 0-9 mg/L). ECG was normal sinus rhythm without any ischaemic changes or changes consistent with pericarditis. Urine dipstick was negative for protein

**Table 1:** Baseline laboratory parameters.

Entity	Results	Normal values with units
Haemoglobin	149	130-180 g/L
White blood cells	8.4	4.0-11.0 109/L
C-reactive protein (CRP)	80	0-9 mg/L
Corrected calcium	2.55	2.20-2.60 mmol/L
CRP	45	0-9 mg/L
Sodium	140	133-146 mmol/L
Alanine aminotransferase (ALT)	8	0-56 µ/L
Alkaline phosphatase (ALP)	77	30-130 U/L
Bilirubin	8	0-21 µmol/L
Lactate dehydrogenase (LDH)	167	135-250 µ/L
Total protein	65	61-78 g/L
Glucose-6-phosphate dehydrogenase (G6PD) screen	Normal	Normal

**Figure 1:**
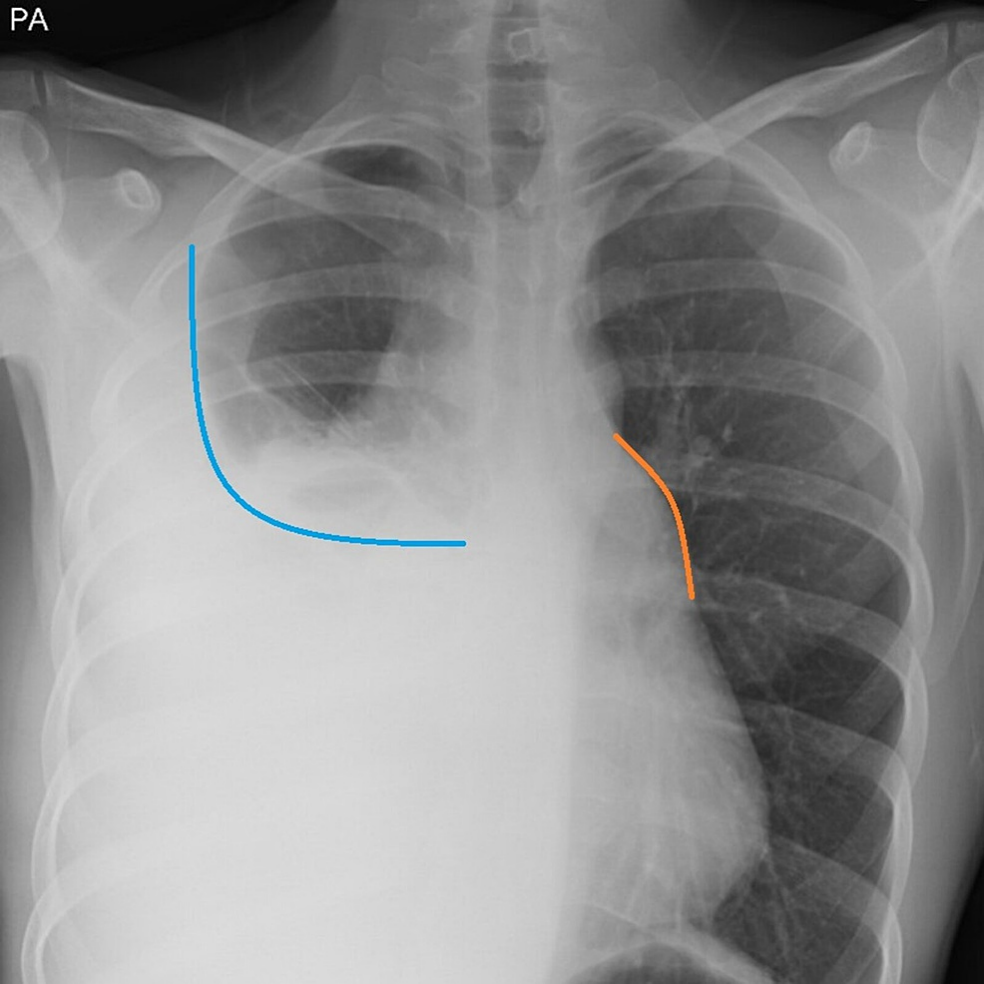
Moderate to large pleural effusion on the right side of the chest (blue curved marking) and mediastinal widening (orange markings).

X-ray chest (Figure 1) showed moderate to large pleural effusion on the right side of the chest with thickening of right paratracheal stripe and mediastinal widening.

We performed a bedside ultrasound of the chest to rule out septations, which were absent. Therefore, he underwent pleural drainage to relieve shortness of breath. The pleural fluid analysis was consistent with transudate Table 2.

**Table 2:** Pleural fluid analysis, which is consistent with transudate.

Entity	Results	Normal values with units
Total protein	24	10-20 g/L
Lactate dehydrogenase (LDH) fluid	194	194 µ/L
Cytology	Mixed inflammatory cells, predominantly neutrophils and macrophages. No malignant cells present	

**Figure 2:**
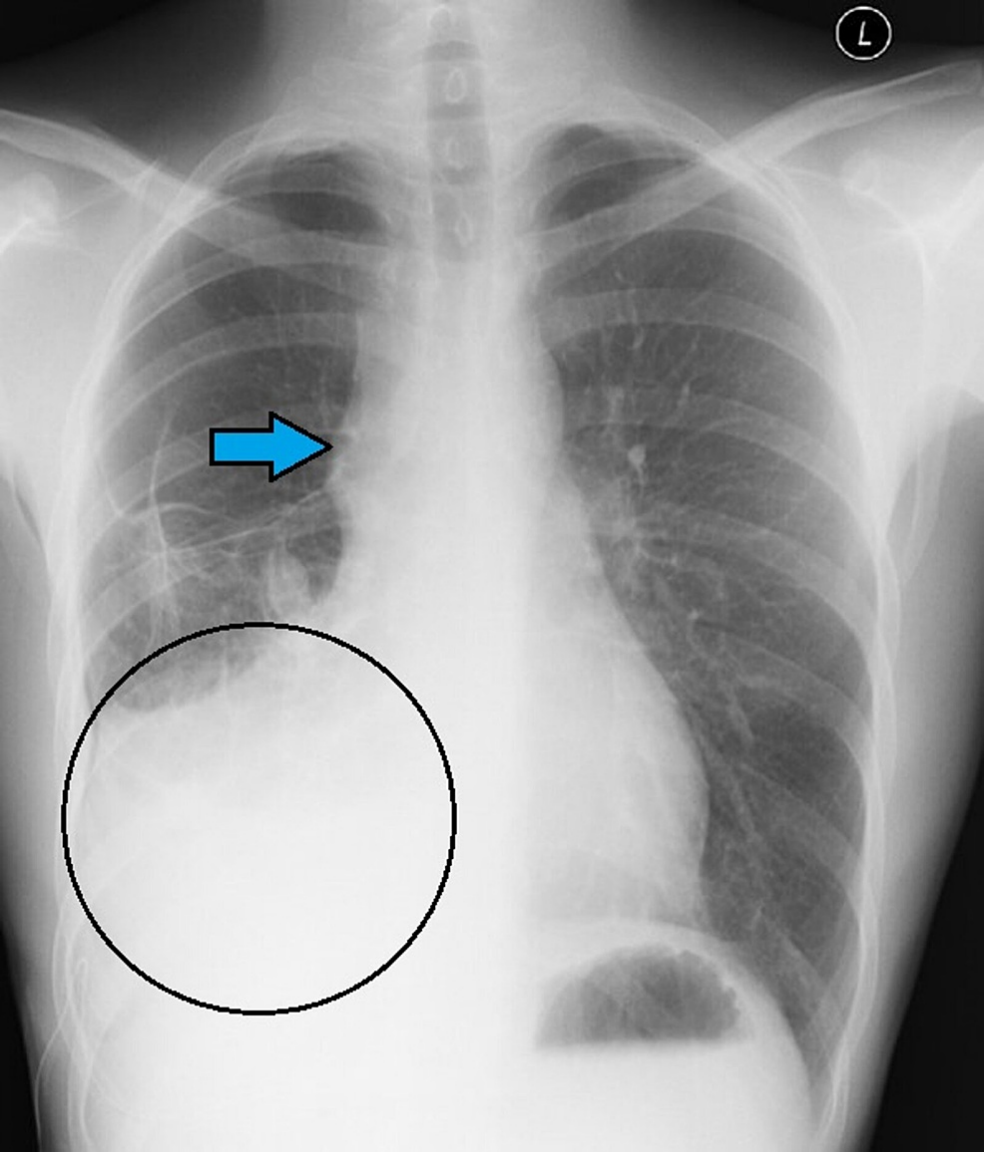
Shows partial resolution of effusion (black circle) and subtle mediastinal enlargement (arrow mark).

Post drainage X-ray chest (Figure 2) showed partial resolution of effusions.

**Figure 3:**
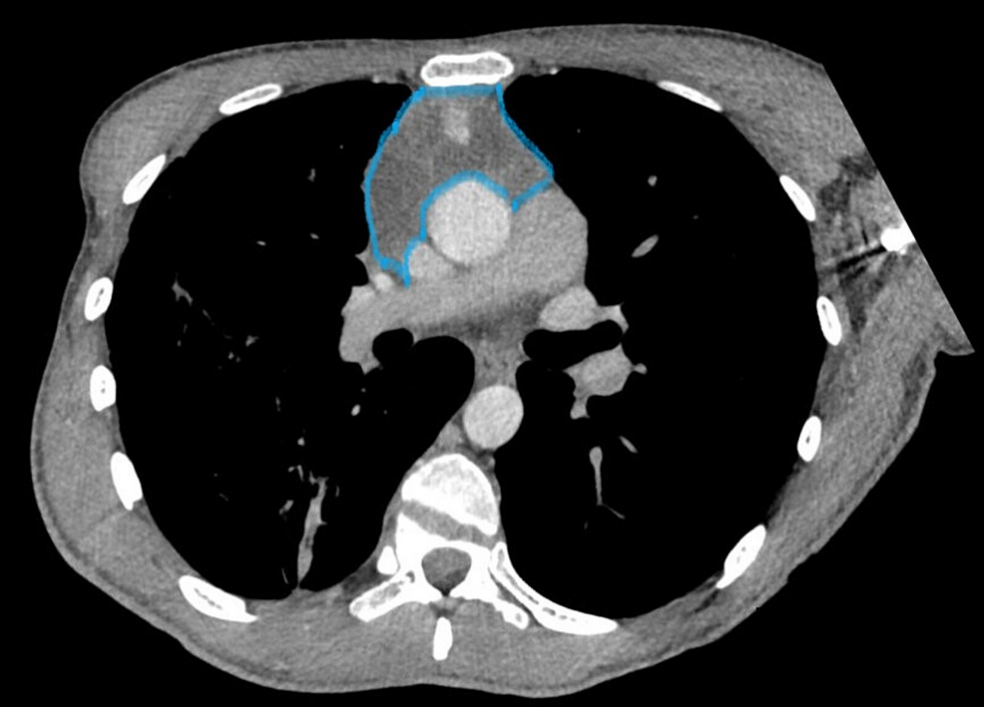
Complex, heterogeneous anterior mediastinal mass, extended into the right hemithorax (marked area).

**Figure 4:**
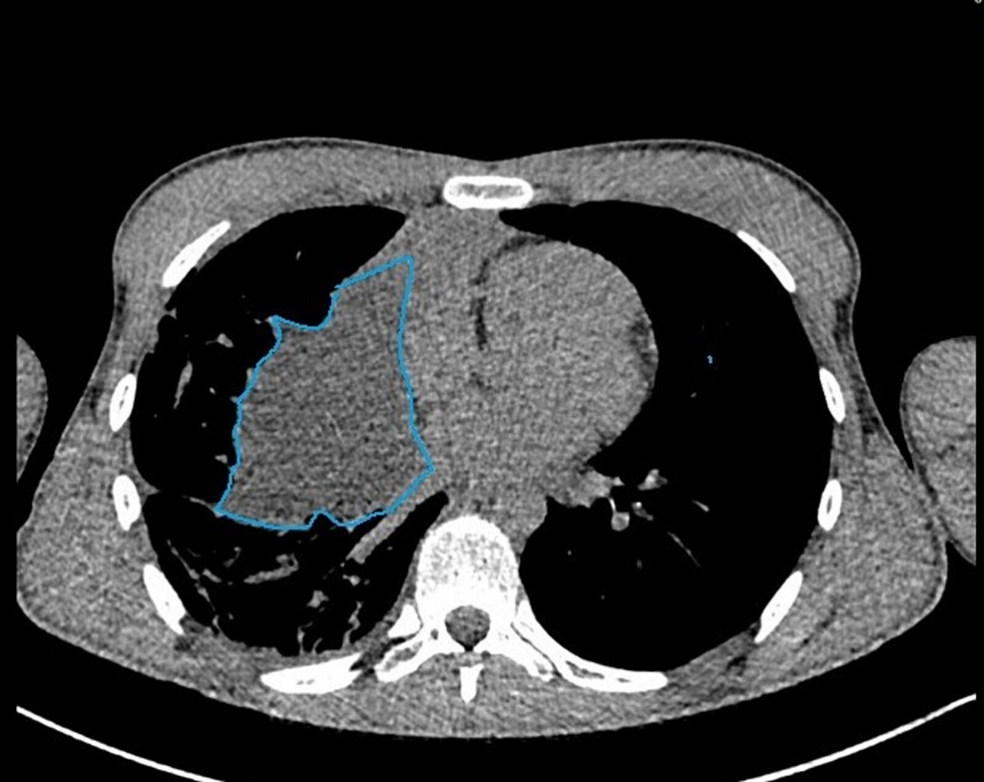
Highlighted area marks the complex mediastinal mass.

We did a CT chest (Figures 3, 4) which demonstrated a complex, heterogeneous anterior mediastinal mass extending into the right hemithorax.

Moreover, there was a possibility that a significant proportion of the right hemithorax fluid on radiographs was part of the cystic extension of the anterior mediastinal lesion. Additionally, tumor markers as shown in Table 3 were negative.

**Table 3:** Illustrates baseline tumor markers.

Entity	Results	Normal range with values
Human chorionic gonadotrophin (B-HCG) IU/L	<1	0-2 IU/L
Alpha-fetoprotein (AFP) kU/L	1.1	0-10.0 kU/L
Beta-2 microglobulin mg/L	2.0	0.8-2.2 mg/L

The patient underwent a CT-guided biopsy, but the sample was not enough to make a diagnosis. Subsequently, the patient underwent an excisional biopsy of the mass on multidisciplinary team advice. 

**Figure 5:**
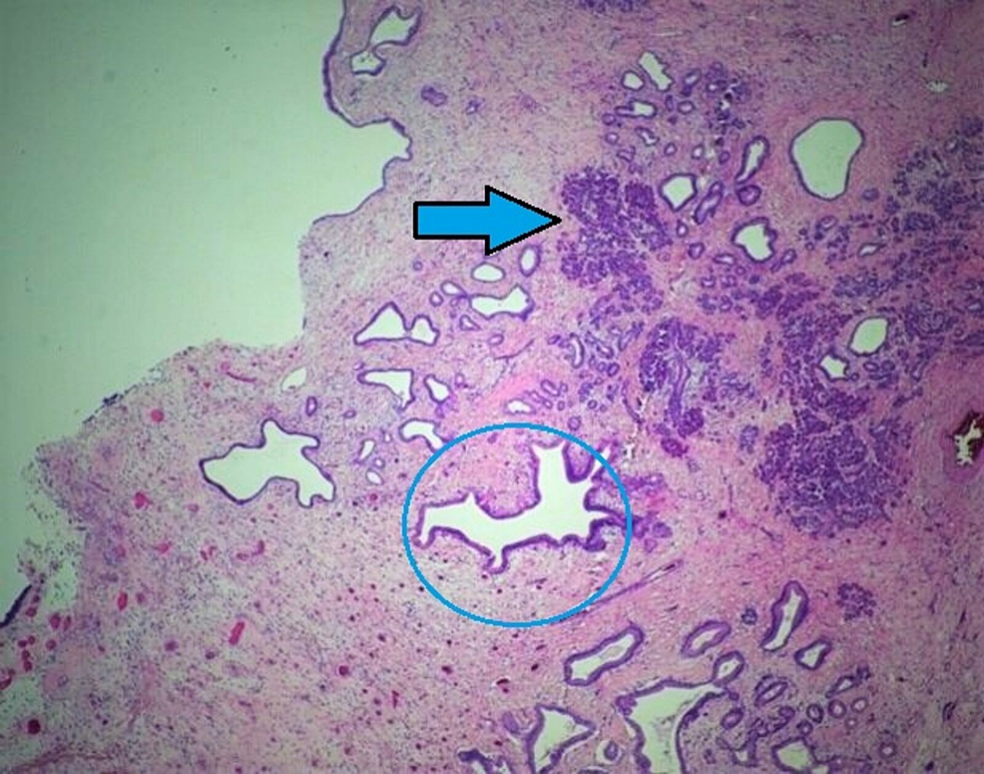
Salivary glands (arrow and circle areas). Haematoxylin and eosin (H&E) x25.

**Figure 6:**
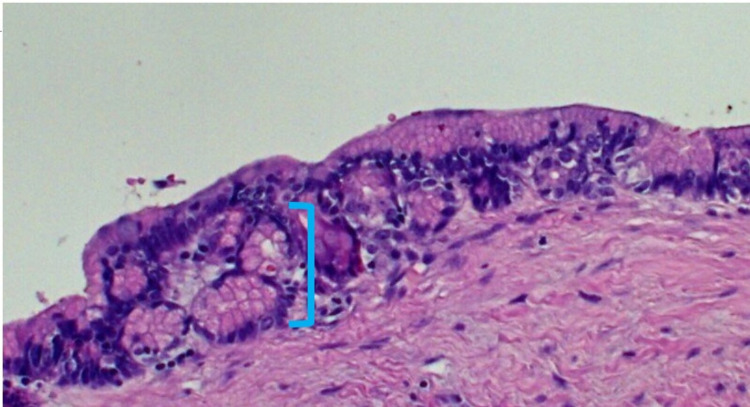
Gastrointestinal epithelium highlighted area. Haematoxylin and eosin (H&E) x100.

**Figure 7:**
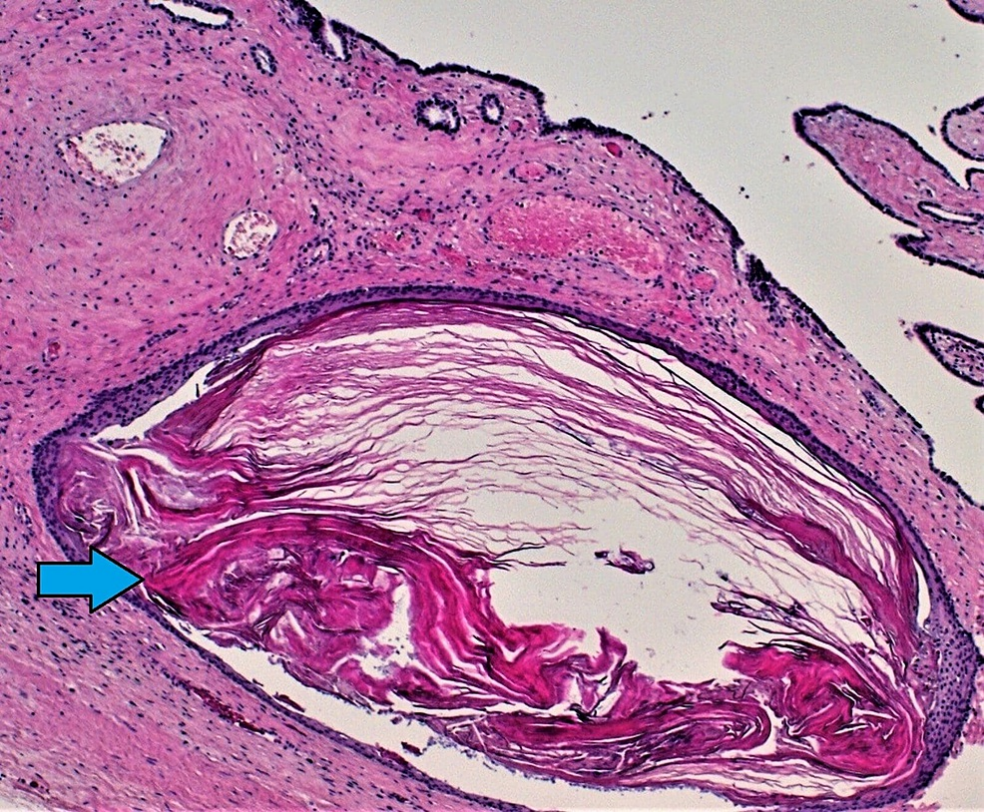
Squamous columnar large nest of pink keratin. Haematoxylin and eosin (H&E) x50.

On the pathological analysis, macroscopically, the tissue sample was well-demarcated approximately 16 x 6 x 13 cm in size and partially encapsulated. Cut sections exhibited irregular arrangement of solid and cystic areas of varying colour and size, including mucoid cysts. Moreover, microscopically, the sections revealed a mature teratoma comprising mature elements, including salivary gland (Figure 5), gastrointestinal (Figure 6) and squamous lined cystic structures (Figure 7). No immature elements or malignancy was seen. Furthermore, the parietal pleura biopsy did not show any evidence of malignancy.

The patient was followed up in the clinic after six weeks postoperatively and remained asymptomatic. Complete surgical removal of mature teratoma is almost always curative and does not require prolonged follow-up; therefore, the patient was discharged from the clinic.

## Discussion

The “teratoma” word originated from the Greek word “teras”, meaning monsters. Teratomas are types of germ cell tumors composed of tissues derived from all three embryonic layers, namely ectoderm, mesoderm and endoderm. They may arise from gonads (ovaries or testes) or maybe extra-gonadal with no primary tumor in ovaries and testes [1]. The anterior mediastinum is the most common extra-gonadal site of germ cell tumors, followed by retroperitoneum and suprasellar regions. Germ cell tumors account for 15% to 16% of all mediastinal tumors, and 50% to 70% of those are teratomas [2].

The exact pathogenesis for the extra-gonadal location of germ cell tumors is unknown. However, a plausible mechanism would be the failure to migrate germ cells to gonadal ridges during embryologic development [3]. Alternatively, there may be reverse migration of germ cells from the gonads to extra-gonadal sites [4].

Generally, teratomas are divided into mature and immature types. Mature teratomas are benign and have equal gender distribution. Immature teratomas, on the other hand, are almost always malignant and exclusively found in males. Mediastinal teratomas are frequently asymptomatic and found incidentally on chest imaging [5]. If present, symptoms are related to mechanical effects of compression and may include cough, breathlessness, and chest pain. Rarely does it present as trichoptysis, which is the result of communication between the tumor and airways [6]. Fistula resulting from pericardial or vascular erosions can have severe consequences, including superior vena cava syndrome or brachial plexus neuritis.

Pleural effusion is rarely seen with teratomas and is only limited to case reports. Moreover, no elucidated data is available regarding the nature of pleural fluid related to teratoma. In our case, effusion was transudative, but in the absence of any obvious etiology and right paratracheal stripe thickening, we decided to do a CT chest that confirmed and characterized anterior mediastinal mass. Later the biopsy established the diagnosis of mature teratoma.

Chest X-ray may show well-defined anterior mediastinal mass with calcification present in 26% of mature teratoma cases [7]. The presence of well-formed teeth or bone is rare on X-ray but highly suggestive of the diagnosis.

CT scan is the investigation of choice, which can accurately locate the mass and characterize different densities within the mass. CT scan can also delineate the relationship with the surrounding structures. The MRI is more helpful to look for the invasion of surrounding structures or in cases of thoracic inlet or thoracoabdominal location of the tumor [8]. Tumor markers (i.e., B-human chorionic gonadotrophin [B-HCG], alpha-fetoprotein [AFP], etc.) are essential if there is an immature component to teratoma or in instances of germ cell tumors other than teratomas.

Differential diagnosis of mediastinal teratomas is vast, usually comprising Thymomas, thyroidal masses, lymphoma and extra-gonadal teratoma. Imaging can help differentiate these masses, but it is prudent to take a tissue biopsy to confirm the diagnosis. Thymomas are usually unilateral with well-defined smooth margins. Microscopically they are composed of lobules of neoplastic epithelial cells and reactive lymphocytes, intersected by fibrous bands. Additionally, they may contain fatty tissue, in which case they are named thymolipomas. Lymphomas present as a diffuse mass, which usually encase mediastinal vessels and have varied histology depending on the type. Thyroidal masses (substernal goitres) usually appear as a continuation of thyroid tissue from the neck, and they may show calcification. On the other hand, teratomas usually exhibit heterogeneous mass with a characteristic display of variable amounts of fat, fluid, soft tissue and intralesional calcification. Intralesional fat is present more frequently; however, fluid that has higher specificity is seen only in fewer cases.

Surgical removal of mediastinal mature teratoma is almost always curative; however, any immature component requires chemotherapy. Mature Teratomas have an excellent prognosis. However, it is vital to make sure that there is no immature component in histopathology. In a study including 45 cases of mature Teratomas, surgical removal resulted in a cure in all the patients [9].

## Conclusions

Mediastinal mature teratoma is a rare and benign condition. Most patients are asymptomatic and rarely present as pleural effusion. Characteristic findings on CT scan and MRI point towards the diagnosis and determine the extent of the disease. The biopsy confirms the diagnosis and rules out any immature elements, which may indicate cancer. In patients with no obvious pathology for pleural effusion, it is prudent to consider teratoma as a potential diagnosis, even if the effusion is transudate. Mature teratoma can be cured by surgical removal.

## References

[1] O'Donovan EJ, Thway K, Moskovic EC (2014). Extragonadal teratomas of the adult abdomen and pelvis: a pictorial review. Br J Radiol.

[2] Travis WD, Brambilla E, Muller-Hermelink HK (2004). Pathology and Genetics of Tumours of the Lung, Pleura, Thymus and Heart. https://patologi.com/who%20lunge.pdf.

[3] Glenn OA, Barkovich AJ (1996). Intracranial germ cell tumors: a comprehensive review of proposed embryologic derivation. Pediatr Neurosurg.

[4] Chaganti RS, Houldsworth J (2000). Genetics and biology of adult human male germ cell tumors. Cancer Res.

[5] Moran CA, Suster S (1997). Primary germ cell tumors of the mediastinum: I. Analysis of 322 cases with special emphasis on teratomatous lesions and a proposal for histopathologic classification and clinical staging. Cancer.

[6] Makarawo TP, Finnikin S, Woolley S, Bishay E (2009). Trichoptysis: a hairy presentation of a rare tumour. Interact Cardiovasc Thorac Surg.

[7] Lewis BD, Hurt RD, Payne WS, Farrow GM, Knapp RH, Muhm JR (1983). Benign teratomas of the mediastinum. J Thorac Cardiovasc Surg.

[8] Sumi A, Nagata S, Zaizen M (2017). Mature cystic teratoma with an element of hepatocellular carcinoma in anterior mediastinum: magnetic resonance-pathologic correlation. J Thorac Imaging.

[9] Dulmet EM, Macchiarin P, Suc B (1993). Germ cell tumors of the mediastinum. A 30-year experience. Cancer.

